# Survival benefits of postoperative radiotherapy in esophageal cancer during the immunotherapy era:a retrospective cohort study based on the SEER database and a single-center registry in China

**DOI:** 10.3389/fimmu.2025.1548520

**Published:** 2025-02-24

**Authors:** Qian Zhang, Tao Zhang, Jiaqi Gu, Xuemei Zhang, Yuxin Mao, Yingying Zhu, Jin Zhang, Jingyi Wang, Shuyang Chen, Yang Cao, Muhong Wang, Chunbo Wang

**Affiliations:** ^1^ Thoracic Radiotherapy, Harbin Medical University Cancer Hospital, Harbin, China; ^2^ The Quzhou Affiliated Hospital of Wenzhou Medical University, Quzhou People’s Hospital, Quzhou, China; ^3^ Department of Oncology, Beidahuang Industry Group General Hospital, Harbin, China

**Keywords:** esophageal cancer, neoadjuvant therapy, immunotherapy, postoperative radiotherapy, SEER database, cohort study

## Abstract

**Purpose:**

The aim of this study was to investigate the survival benefits of postoperative radiotherapy (PORT) in patients with resectable esophageal cancer (EC) after neoadjuvant therapy in the Immunotherapy era.

**Methods:**

The study was designed as a retrospective cohort study, which included a total of 733 patients with EC from the SEER database and a single-center cohort. We used propensity score matching (PSM) to equilibrate patient characteristics. The investigation incorporated Kaplan-Meier survival analysis and the Cox proportional risk regression model to assess outcomes.

**Results:**

PORT did not significantly improve survival in the overall cohort, with a median overall survival of 38 months (p=0.56) in the SEER cohort and 39 months (p=0.75) in the Chinese cohort. However, in the immunotherapy subgroup, the Chinese cohort demonstrated that immunotherapy combined with PORT significantly improved survival (p=0.044).Multivariate Cox regression analysis demonstrated that patients aged 50-59 years (HR=5.93, 95% CI: 1.67-21.06) and those aged ≥70 years (HR=10.96, 95% CI:3.04-39.56) had increased survival risks compared to patients aged <50 years. Additionally, ypT3-4 stage patients exhibited a higher risk than those with ypT1-2 stage (HR=2.12, 95% CI: 1.14-3.93, p=0.017).Similar trends were observed in cT3-4 staging, R1/R2 and no immunotherapy. Lymph node metastasis also showed a progressive relationship with survival risk, with patients categorized as ypN1 (HR=1.90), ypN2 (HR=4.24), and ypN3 (HR=6.68) experiencing increasingly higher risks (p<0.05).

**Conclusions:**

The collaborative effect of immunotherapy and PORT potentially enhances survival outcomes for patients with EC. However, further prospective research is essential to confirm our results.

## 1 Introduction

According to GLOBOCAN 2022 statistics, esophageal cancer (EC) ranks as the seventh deadliest malignant neoplasm globally, contributing to 4.6% of all cancer-related fatalities, and its incidence is particularly prominent in Asian countries, such as China, where more than 70% of patients are male (1). For locally advanced resectable EC, the integration of neoadjuvant chemoradiation therapy (nCRT) followed by surgical intervention has emerged as the standard treatment approach, and pivotal clinical trials, such as CROSS and NEOCRTEC-5010, have shown notable enhancements in both overall survival (OS) and disease-free survival (DFS) (2, 3).

Although neoadjuvant therapy has improved the surgical resection rate and patient prognosis, postoperative recurrence and distant metastasis remain major clinical challenges, with the postoperative recurrence rate remaining as high as 31% even after nCRT and the surgical recurrence rate alone being as high as 49% (4, 5). Moreover, the clinical application of neoadjuvant therapy still faces many challenges, and patient tolerance and treatment-related adverse effects are key factors that restrict its development (6, 7). Therefore, it is crucial to develop an optimal postoperative treatment strategy; however, the results of existing studies are divided according to whether postoperative radiotherapy (PORT) can provide a survival benefit (8, 9).

Since the approval of Immune Checkpoint Inhibitors (ICIs) for EC treatment in 2019, the treatment paradigm has undergone fundamental changes. Studies have shown that immunotherapy not only significantly prolongs survival in advanced patients (10) but also demonstrates great potential in neoadjuvant and adjuvant therapy (11, 12). Immunotherapy combined with radiotherapy has potential synergistic effects and activates systemic anti-tumor immune responses (13). However, the value of PORT after neoadjuvant therapy and synergistic effects of immuno-combination radiotherapy have not been fully validated in the era of immunotherapy. Consequently, this research sought to assess the impact of PORT after neoadjuvant treatment on the prognosis of patients and provide a new basis for optimizing treatment strategies.

## 2 Materials and methods

### 2.1 Patients

This was a two-cohort, retrospective study. The first cohort dataset was obtained from the Affiliated Cancer Hospital of Harbin Medical University (January 2014 to October 2024). The second cohort data were obtained from SEER Research Data 17 Registries November 2023 (January 2004 to December 2015 and January 2019 to December 2021) via SEER*Stat software (version 8.4.4) and patients who received immunotherapy based on the time when it was approved (2019) for immunotherapy patients.

**Figure 1:**
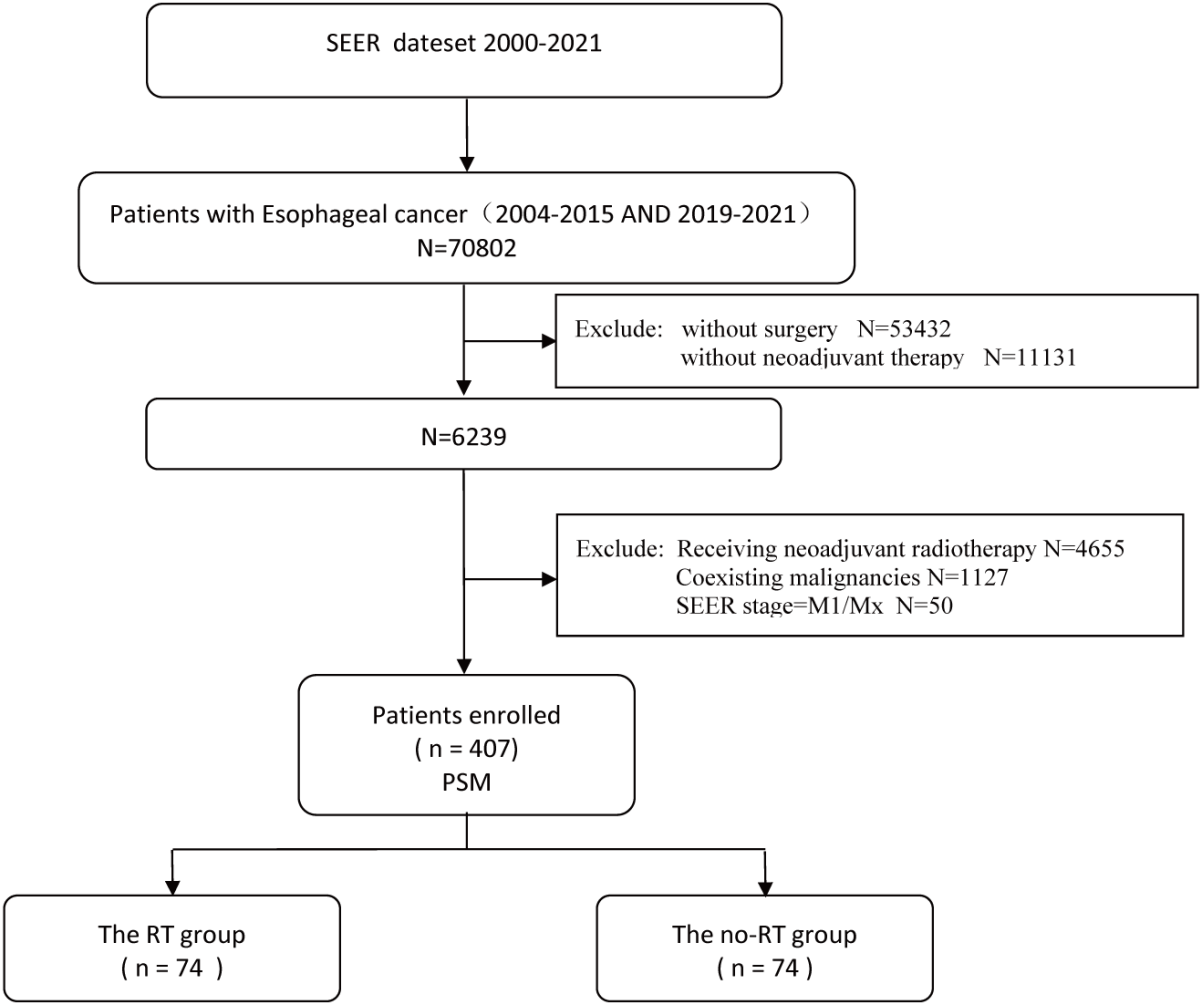
Flowchart of the patients screening process.

The inclusion criteria were (1) pathologically confirmed EC (ICD-O-3/WHO 2008 classification), (2) preoperative systemic therapy without radiotherapy, and (3) clinical stage cM0 (based on the AJCC/UICC 8th edition staging criteria). The exclusion criteria were (1) non-primary esophageal malignancy, (2) unknown surgical status, and (3) incomplete clinical data. The patient screening process is illustrated in **Figure 1**.

The criteria of the PORT:patients with R1 or R2 resections who did not receive nCRT, as well as R0 resected N+ or pT3-4aN0 patients, were generally recommended for PORT. For adenocarcinoma (AC) patients, high-risk pT2N0 patients were also advised to receive radiotherapy. In actual clinical practice, radiation oncologists comprehensively evaluate surgical conditions, postoperative imaging findings, recurrence risk, and high-risk factors (such as poor differentiation, lymphovascular invasion, perineural invasion, and age <50 years) to personalize radiotherapy treatment plans.

The clinical information and data used in the study of the single-center cohort were exempted from informed consent and ethical approval owing to the minimal risk of using an electronic medical record system. The SEER database is publicly available and does not require additional ethical approval.

### 2.2 Treatment protocols

In the Chinese cohort of this study, all patients in the immunotherapy group received neoadjuvant chemoimmunotherapy, with 28% subsequently undergoing postoperative chemoimmunotherapy. The treatment regimen specifically involved intravenous administration of 200 mg Camrelizumab, Sintilimab, or Pembrolizumab every 3 weeks, with a typical treatment course of 2-4 cycles. In contrast, patients not receiving immunotherapy underwent at least 2 cycles of conventional platinum-based chemotherapy, including Cisplatin, Carboplatin, or Nedaplatin, combined with Taxanes (Docetaxel or Paclitaxel) and Tegafur. Notably, all drug dosages were individually adjusted by the attending physicians based on the patients' specific body surface area, physical condition, and tolerability to optimize treatment efficacy and minimize adverse reactions.

Radiotherapy precisely covered the post-operative tumor bed and high-risk lymph node drainage areas, with a total dose of 50.4-60 Gy. For patients with post-operative residual or suspicious lymph nodes, a curative dose could be further applied. Radiation was delivered at 1.8-2 Gy per fraction, 5 times per week, using techniques including three-dimensional conformal radiotherapy (3D-CRT) and intensity-modulated radiotherapy (IMRT). To optimize treatment outcomes and patient prognosis, esophagectomy was typically performed 4-8 weeks after neoadjuvant therapy, ensuring adequate treatment intervals and bodily recovery.

### 2.3 Statistical analysis

To compare the initial characteristics between the RT and no-RT groups, researchers employed both the chi-square test and Fisher's exact test. Using the MaxStat method, we identified the ideal cut-off value for TRR(Tumor regression rate,%). Propensity score matching (PSM) analysis was conducted on patients from both cohorts to equalize group characteristics. The analysis utilized a 1:1 ratio and the nearest neighbor matching approach. Covariates exhibiting p<0.05 or SMD>0.1 were included in the matching process for both groups. (In the SEER cohort, matching variables included immunotherapy status, sex, age, T stage, and N stage, whereas the Chinese cohort's matching variables encompassed a more comprehensive set, including immunotherapy status, sex, age, pathology, histologic grade, primary site, T stage, and N stage). To evaluate overall survival (OS) and cancer-specific survival (CSS) rates, we employed the Kaplan-Meier method. Survival differences were assessed using the log-rank test. Univariate and multivariate Cox proportional hazard regression models were employed to the evaluate factors influencing OS prognosis. Variables with p ≤0.05 in the univariate analysis were subsequently incorporated into the multivariate analysis. R software (version 4.3.2) was utilized for all statistical computations, with statistical significance defined as a two-sided p-value<0.05.

## 3 Results

### 3.1 Clinical characteristics

The study included 407 individuals from the SEER cohort and 326 individuals from the Chinese cohort. In terms of demographic characteristics, patients in the SEER cohort were mainly concentrated in the age group of 60-69 years (no-RT 37.54% vs. RT 50.00%, p=0.089), with an overwhelming majority of males (no-RT 80.18% vs. RT 86.49%, p=0.274). Oncological features showed that lower esophagus (no-RT 74.47% vs. RT 77.03%, p=0.887) and AC (no-RT 78.98% vs. RT 78.38%, p=0.895) were the main types. Immunotherapy application rates were 25.83% (no-RT) and 20.27% (RT) (p=0.394). The Chinese cohort showed significant regional specificity in terms of the demographic and oncological characteristics. Patients were predominantly 50-59 years old (no-RT 43.67% vs. RT 41.24%, p=0.048), and the proportion of males was nearly 100% (no-RT 95.20% vs. RT 98.97%, p=0.183). Notably, squamous cell carcinoma (SCC) was dominant (no RT 93.89% vs. RT 96.91%, p=0.532). Compared to the SEER cohort, the application rate of immunotherapy has increased (no RT 63.32% vs. RT 46.39%, p=0.007). After PSM, there were 148 cases in the SEER database cohort (74 [50%] in the no-RT group and 74 [50%] in the RT group) and 194 cases in the Chinese cohort (97 [50%] in the no-RT group and 97 [50%] in the RT group). The two cohort groups exhibited no statistically significant differences in baseline characteristics. **Tables 1** and **2** show the baseline clinicopathological features of the study population before and after PSM.

**Table 1:** Baseline characteristics of patients included in the analysis before PSM.

Characteristics	SEER cohort			Single-Center Cohort		
	Non-PORT	PORT	p-value	Non-PORT	PORT	p-value
	(N,%)	(N,%)		(N,%)	(N,%)	
Total	333 (81.80)	74 (18.20)		229 (70.20)	97 (29.80)	
Age			0.089			0.048
<50	27 (8.11)	5 (6.76)		21 (9.17)	5 (5.15)	
50-59	72 (21.62)	18 (24.32)		100 (43.67)	40 (41.24)	
60-69	125 (37.54)	37 (50.00)		92 (40.17)	36 (37.11)	
≥70	109 (32.73)	14 (18.92)		16 (6.99)	16 (16.49)	
Sex			0.274			0.183
Male	267 (80.18)	64 (86.49)		218 (95.20)	96 (98.97)	
Female	66 (19.82)	10 (13.51)		11 (4.80)	1 (1.03)	
Race			0.589			
White	298 (89.49)	64 (86.49)				
Balck and others	35 (10.51)	10 (13.51)				
TRR						0.987
<0.29				76 (33.19)	33 (34.02)	
≥0.29				153 (66.81)	64 (65.98)	
Surgical technique						0.012
Sweet				15 (6.55)	7 (7.22)	
Ivor-Lewis				40 (17.47)	31 (31.96)	
McKeown				174 (75.98)	59 (60.82)	
Histologic grade						0.322
I				67 (29.26)	36 (37.11)	
II				70 (30.57)	29 (29.90)	
III				92 (40.17)	32 (32.99)	
Primarysite			0.887			0.66
Middle and upper	43 (12.91)	9 (12.16)		30 (30.93)	36 (37.11)	
lower	248 (74.47)	57 (77.03)		26 (26.80)	24 (24.74)	
Overlapping or other	42 (12.61)	8 (10.81)		41 (42.27)	37 (38.14)	
Histology			0.895			0.532
Adenocarinoma	263 (78.98)	58 (78.38)		9 (3.93)	2 (2.06)	
SCC	58 (17.42)	14 (18.92)		215 (93.89)	94 (96.91)	
other	12 (3.60)	2 (2.70)		5 (2.18)	1 (1.03)	
T stage*			0.202			0.06
ypT1-2	93 (27.93)	16 (21.62)		113 (49.34)	43 (44.33)	
ypT3-4	125 (37.54)	36 (48.65)		102 (44.54)	53 (54.64)	
ypTx	115 (34.53)	22 (29.73)		14 (6.11)	1 (1.03)	
N stage*			0.354			<0.001
ypN0	173 (51.95)	33 (44.59)		114 (49.78)	25 (25.77)	
ypN1	60 (18.02)	21 (28.38)		74 (32.31)	45 (46.39)	
ypN2	29 (8.71)	7 (9.46)		25 (10.92)	20 (20.62)	
ypN3	24 (7.21)	4 (5.41)		16 (6.99)	7 (7.22)	
ypNx	47 (14.11)	9 (12.16)				
Immunotherapy			0.394			0.007
Yes	86 (25.83)	15 (20.27)		145 (63.32)	45 (46.39)	
No	247 (74.17)	59 (79.73)		84 (36.68)	52 (53.61)	

Tumor Reduction Rate (TRR) = (Maximum Tumor Diameter before Treatment - Tumor Diameter at the Same Location after Treatment) / Maximum Tumor Diameter before Treatment × 100%

TRR was calculated based on CT images, using the maximum tumor diameter before treatment as the reference, and precisely measuring the tumor diameter at the same anatomical location after treatment to ensure measurement accuracy and consistency.

PORT, postoperative radiotherapy; T, tumor; N, nodal; SCC, squamous cell carcinoma;

*The staging provided in the database was further subdivided into yp and c stages based on the “CS Lymph Nodes Eval (2004–2015)”;”CS Tumor Size/Ext Eval (2004-2015)”;”Regional nodes positive (1988+)”field extracted from the SEER data.

**Table 2:** Baseline characteristics of patients included in the analysis after PSM.

Characteristics	SEER cohort			Single-Center Cohort		
	Non-PORT	PORT	p-value	Non-PORT	PORT	p-value
	(N,%)	(N,%)		(N,%)	(N,%)	
Total	74 (50.00)	74 (50.00)		97 (50.00)	97 (50.00)	
Age			0.960			0.526
<50	6 (8.11)	5 (6.76)		7 (7.22)	5 (5.15)	
50-59	16 (21.62)	18 (24.32)		38 (39.18)	40 (41.24)	
60-69	39 (52.70)	37 (50.00)		42 (43.30)	36 (37.11)	
≥70	13 (17.57)	14 (18.92)		10 (10.31)	16 (16.49)	
Sex			1			1
Male	65 (87.84)	64 (86.49)		96 (98.97)	96 (98.97)	
Female	9 (12.16)	10 (13.51)		1 (1.03)	1 (1.03)	
Race			0.606			
White	67 (90.54)	64 (86.49)				
Balck and others	7 (9.46)	10 (13.51)				
TRR						1
<0.29				32 (32.99)	33 (34.02)	
≥0.29				65 (67.01)	64 (65.98)	
Surgical technique						0.338
Sweet				9 (9.28)	7 (7.22)	
Ivor-Lewis				22 (22.68)	31 (31.96)	
McKeown				66 (68.04)	59 (60.82)	
Histologic grade						0.781
I				32 (32.99)	36 (37.11)	
II				33 (34.02)	29 (29.90)	
III				32 (32.99)	32 (32.99)	
Primarysite			0.467			0.660
Middle and upper	7 (9.46)	9 (12.16)		30 (30.93)	36 (37.11)	
lower	54 (72.97)	57 (77.03)		26 (26.80)	24 (24.74)	
Overlapping or other	13 (17.57)	8 (10.81)		41 (42.27)	37 (38.14)	
Histology			0.834			0.902
Adenocarinoma	59 (79.73)	58 (78.38)		3 (3.09)	2 (2.06)	
SCC	12 (16.22)	14 (18.92)		93 (95.88)	94 (96.91)	
other	3 (4.05)	2 (2.70)		1 (1.03)	1 (1.03)	
T stage*			0.693			0.674
ypT1-2	15 (20.27)	16 (21.62)		38 (39.18)	43 (44.33)	
ypT3-4	35 (47.30)	36 (48.65)		57 (58.76)	53 (54.64)	
ypTx	24 (32.43)	13 (29.73)		2 (2.06)	1 (1.03)	
N stage*			0.784			0.623
ypN0	37 (50.00)	33 (44.59)		30 (30.93)	25 (25.77)	
ypN1	22 (29.73)	21 (28.38)		37 (38.14)	45 (46.39)	
ypN2	4 (5.41)	7 (9.46)		20 (20.62)	20 (20.62)	
ypN3	2 (2.70)	4 (5.41)		10 (10.31)	7 (7.22)	
ypNx	9 (12.16)	9 (12.16)				
Immunotherapy			0.439			1
Yes	20 (27.03)	15 (20.27)		45 (46.39)	45 (46.39)	
No	54 (72.97)	59 (79.73)		52 (53.61)	52 (53.61)	

*The staging provided in the database was further subdivided into yp and c stages based on the “CS Lymph Nodes Eval (2004–2015)”;”CS Tumor Size/Ext Eval (2004-2015)”;”Regional nodes positive (1988+)”field extracted from the SEER data.

### 3.2 Survival analysis

**Figures 2:**
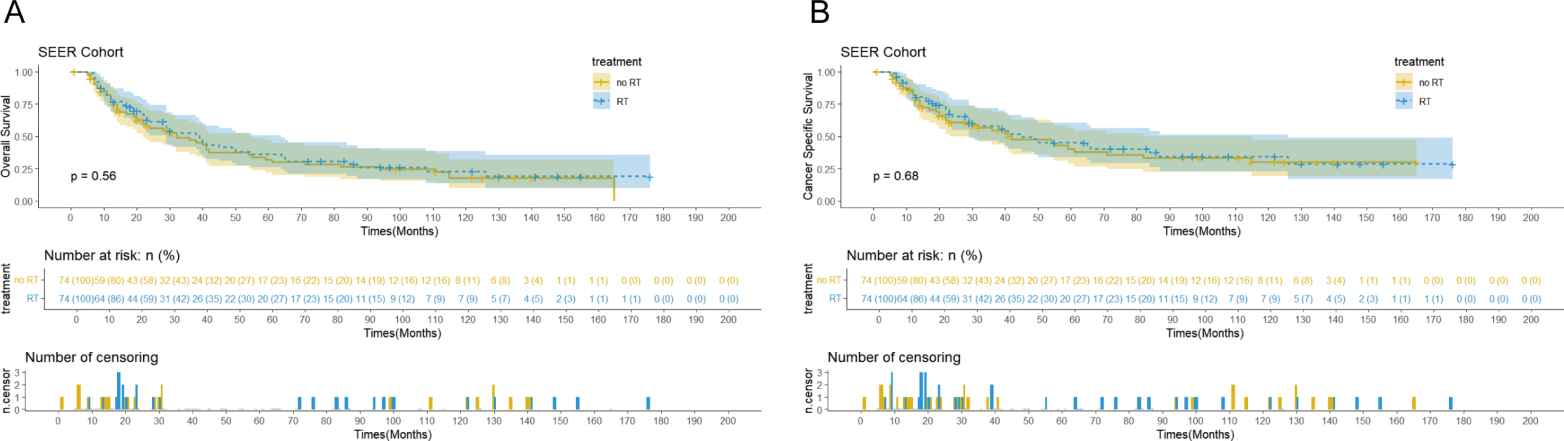
Kaplan-Meier curves of overall survival (OS) **(A)** and cancer-specific survival (CSS) **(B)** for patients with and without postoperative radiotherapy (PORT) in theSEER cohort after propensity score matching (PSM).

**Figures 3:**
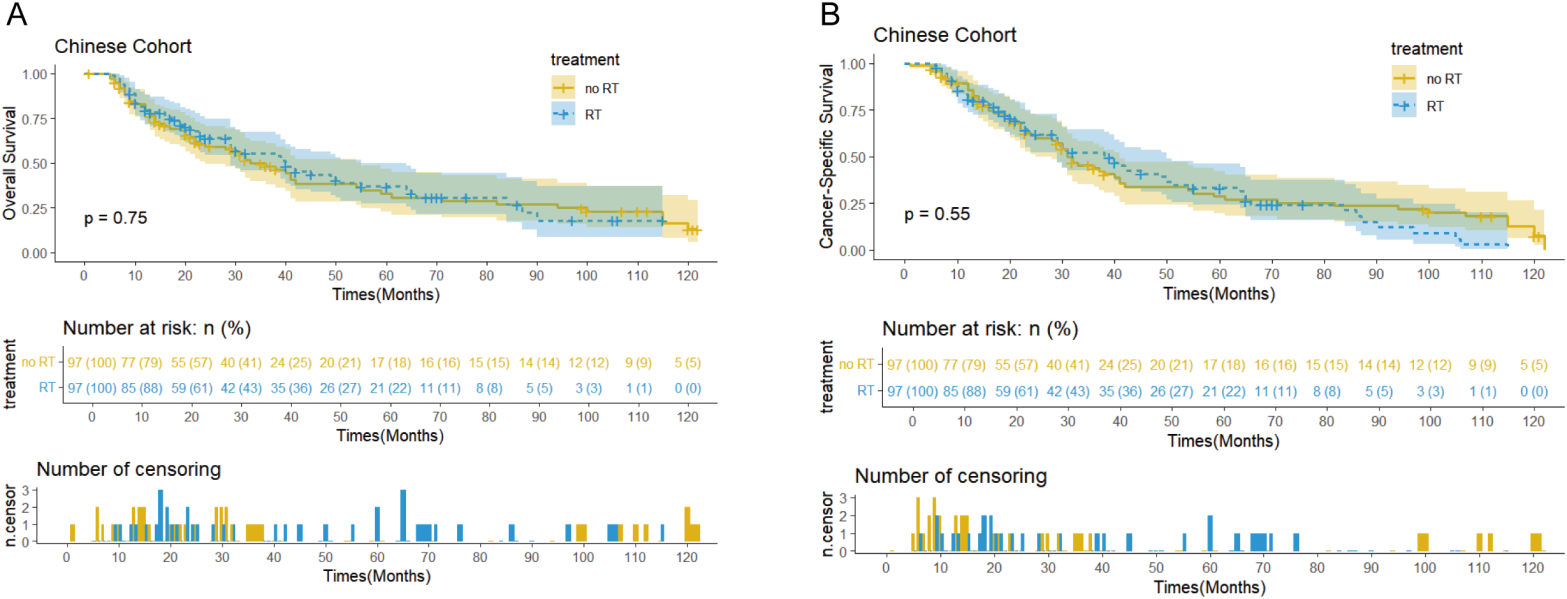
Kaplan-Meier curves of OS **(A)** and CSS **(B)** for patients with and without PORT in the Chinese cohort after PSM.

The SEER cohort had a median follow-up of 122 months (interquartile range: 31-140 months) and median OS of 38 months (95% confidence interval: 29-45 months). In terms of disease-specific survival, the median CSS was 42 months (95% confidence interval: 32-71 months, **Figures 2A, B**). In comparison, the Chinese cohort showed similar results, with a median follow-up of 65 months (interquartile range: 29-107 months), median OS of 39 months (95% confidence interval: 30-49 months), and overall median CSS of 32 months (95% confidence interval: 29-41 months, **Figures 3A, B**).

**Figures 4:**
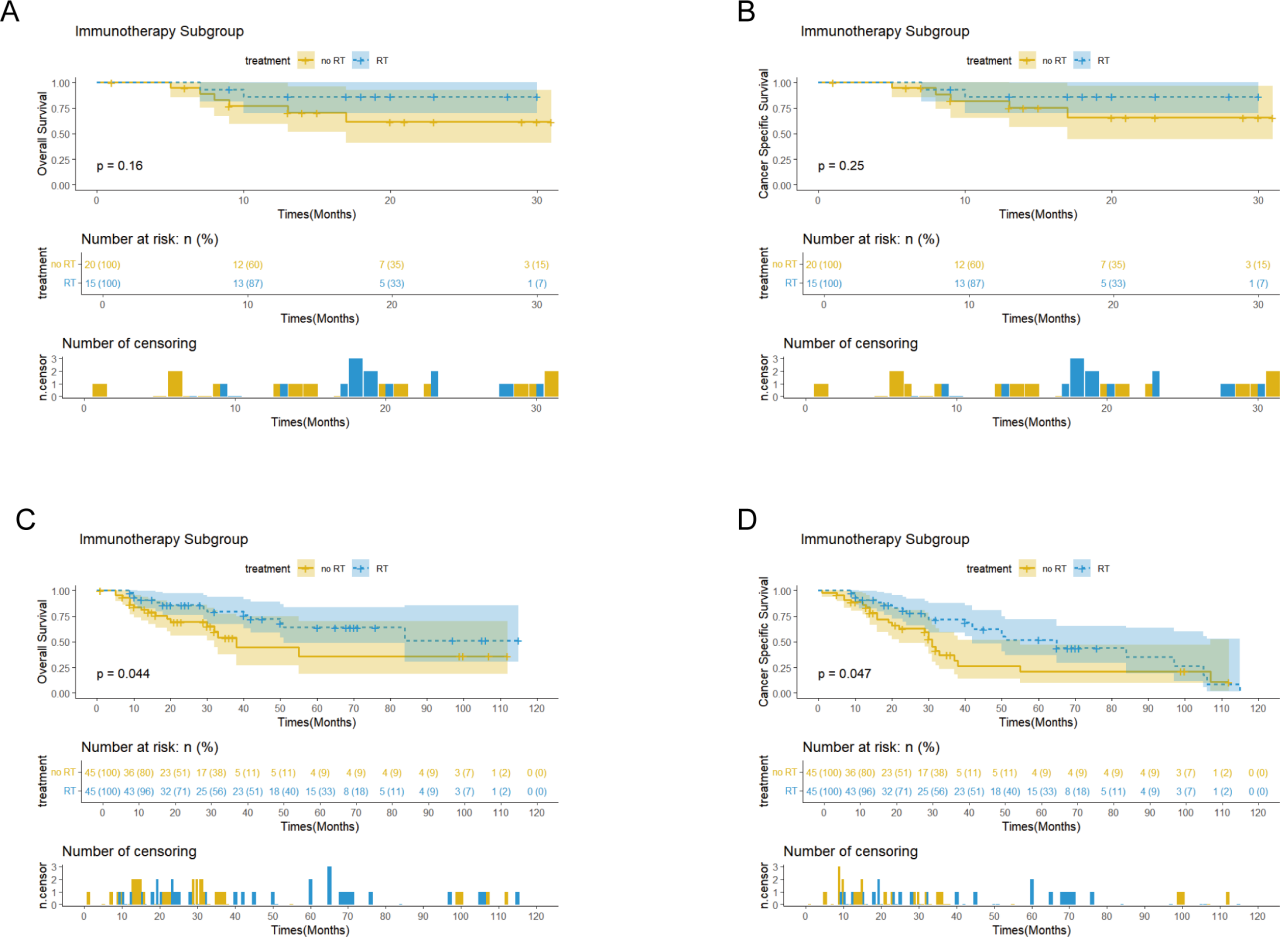
Kaplan-Meier curves of OS and CSS for patients with and without PORT (RT&no RT) in the SEER **(A, B)** and Chinese **(C, D)** cohorts, stratified by immunotherapy status after PSM.

**Figures 5:**
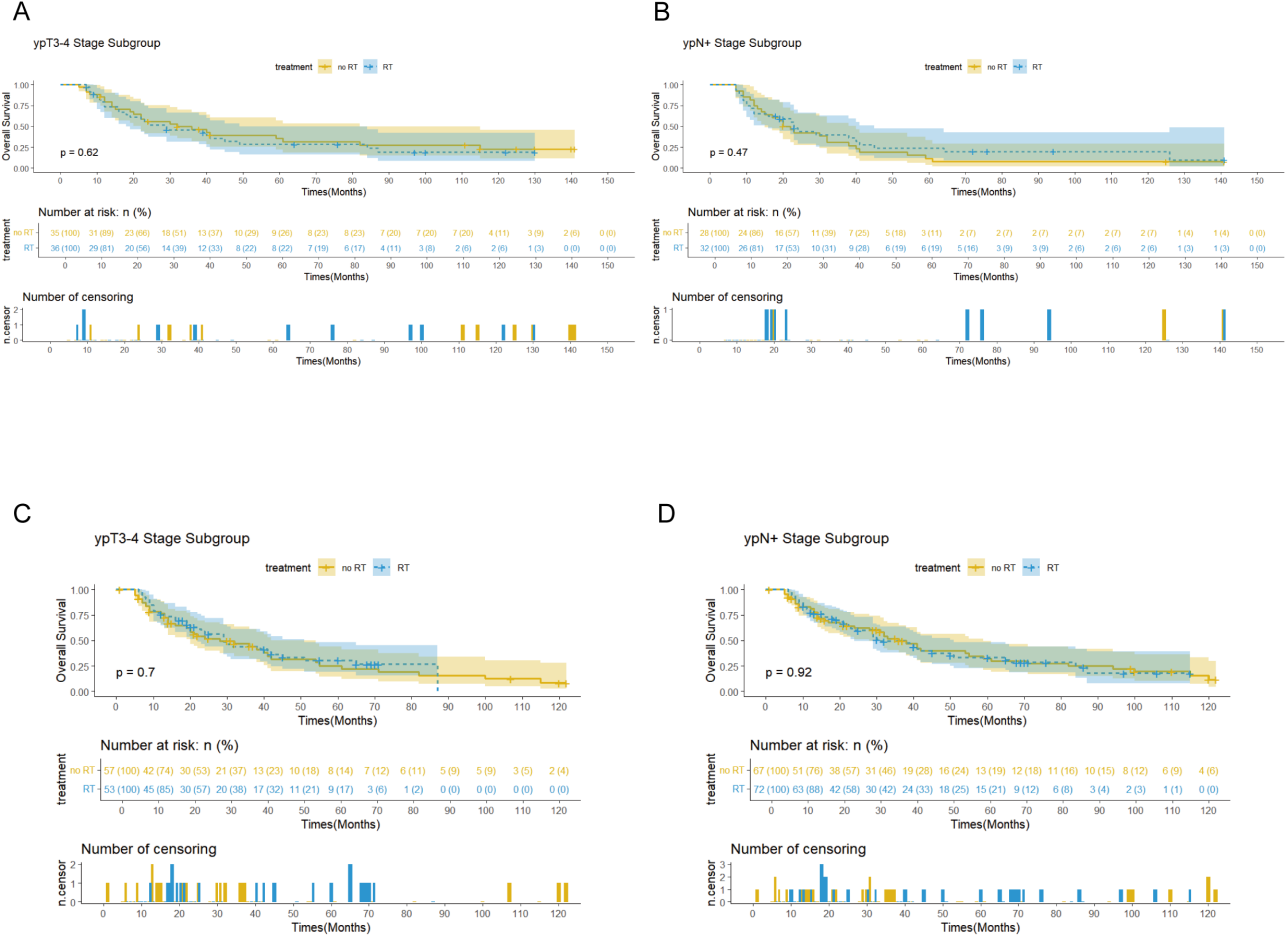
Survival curves showed the OS for the patients with ypT3-4 stage and ypN+ stage in the SEER **(A, B)** and Chinese **(C, D)** cohorts.

Survival analysis showed that PORT did not significantly improve OS (p=0.56 vs 0.75) or CSS (p=0.68 vs 0.55) in patients with EC. Notably, the immunotherapy subgroup analysis revealed differences between cohorts. In the Chinese cohort, PORT demonstrated a statistically significant enhancement in OS (p= 0.044) and CSS (p= 0.047), as shown in **Figures 4C, D**, with median OS and CSS not achieved in either group. However, no similar survival advantage was observed in the SEER cohort study (**Figures 4A, B**). Further subgroup analyses based on ypT3-4 stage, ypN+ status, and resection status failed to confirm a survival benefit of PORT even among these high-risk subgroups of patients (**Figures 5A–D**, **Supplementary Figure 2**). Furthermore, detailed stratified survival analysis of squamous cell carcinoma patients in both the SEER and Chinese cohorts failed to demonstrate significant survival improvements from PORT (**Supplementary Figure 1**).

**Table 3** presents the factors influencing OS in both univariate and multivariate analyses for the two matched cohorts. In the SEER cohort, age significantly affected patient survival. Compared to the reference group aged <50 years, patients in the 50-59 years (HR=2.71, 95%CI: 1.04-7.08, p=0.041) and ≥70 years (HR=3.99, 95%CI: 1.5-10.62, p=0.006) exhibited statistically significant elevated hazard ratios for survival. In contrast, age did not have a significant effect on prognosis in the Chinese cohort, suggesting that there may be differences in biological behavior and prognosis across populations and geographic regions. Tumor stage was a common prognostic factor in both cohorts. Patients with ypT3-4 stage had a significantly higher survival risk than those with ypT1-2 stage in the SEER cohort (HR=2.21, 95%CI: 1.32-3.71, p=0.003); similar findings were noted in the Chinese cohort (HR=1.86, 95%CI: 1.29-2.67, p=0.001). This concordance further validates the generalized impact of tumor T-staging on EC prognosis. The two cohorts exhibited notable disparities in the occurrence of lymph node metastasis. The SEER cohort showed a clear incremental stage-risk relationship; the survival risk was progressively higher in stages N1 (HR=2.05, p=0.004), N2 (HR=2.65, p=0.007), and N3 (HR=6.37, p<0.01). In contrast, statistical significance of lymph node metastasis as a prognostic factor was not observed in the Chinese cohort.

**Table 3:** Univariable and multivariable Cox regression analyses for overall survival of patients after PSM.

Characteristics	Univariate				Multivariate			
	SEER Cohort		Single-Center Cohort		SEER Cohort		Single-Center Cohort	
	HR/CI^1^	P	HR/CI^1^	P	HR/CI^1^	P	HR/CI^1^	P
Age								
<50	1		1		1		1	
50-59	2.71 (1.04-7.08)	0.041	1.93 (0.83-4.48)	0.128	5.93 (1.67 - 21.06)	0.006		
60-69	1.93 (0.76-4.89)	0.164	1.63 (0.69-3.84)	0.262				
≥70	3.99 (1.5-10.62)	0.006	1.35 (0.51-3.62)	0.547	10.96 (3.04 - 39.56)	<0.01		
Sex								
Male	1		1					
Female	0.91 (0.47-1.76)	0.783	0 (0-Inf)	0.995				
Race								
White	1							
Black and others	1.33 (0.73-2.44)	0.356						
TRR								
<0.29			1					
≥0.29			0.77 (0.52-1.14)	0.197				
Surgical technique								
Sweet			1					
Ivor-Lewis			1.05 (0.56-1.96)	0.872				
McKeown			0.67 (0.37-1.2)	0.175				
Histologic grade								
I			1					
II			0.97 (0.63-1.49)	0.885				
III			0.68 (0.43-1.07)	0.097				
Primarysite								
Middle and upper	1		1					
lower	0.88 (0.46-1.71)	0.716	0.79 (0.48-1.3)	0.345				
Overlapping or other	1.36 (0.6-3.06)	0.463	0.02 (0.68-1.54)	0.912				
Histology								
Adenocarinoma	1		1					
SCC	1.27 (0.75-2.16)	0.371	0.85 (0.31-2.3)	0.743				
other	0.53 (0.13-2.16)	0.375	2.24 (0.41-12.37)	0.354				
T stage								
ypT1-2	1		1		1			
ypT3-4	2.21 (1.32-3.71)	0.003	1.86 (1.29-2.67)	0.001	2.12 (1.14 - 3.93)	0.017	1.72 (1.15 - 2.85)	0.012
ypTx	1.50 (0.77-2.93)	0.238	–	–				
N stage								
ypN0	1		1		1		1	
ypN1	2.05 (1.26-3.32)	0.004	1.11 (0.7-1.76)	0.658	1.90 (1.12 - 3.2)	0.021		
ypN2	2.65 (1.31-5.39)	0.007	1.27 (0.74-2.17)	0.389	4.24 (1.74 - 10.33)	0.001		
ypN3	6.37 (2.59-15.71)	<0.01	1.76 (0.91-3.4)	0.093	6.68 (2.43 - 18.32)	<0.01		
ypNx	2.06 (1.12-3.79)	0.02			2.78 (1.30 - 5.95)	<0.01		
Immunotherapy								
Yes	1		1		1		1	
No	1.51 (0.71-3.18)	0.281	2.59 (1.71-3.94)	<0.01			2.87 (1.76 - 4.69)	<0.01
PORT								
No	1		1		1		1	
Yes	0.89 (0.6-1.32)	0.567	0.94 (0.65-1.36)	0.745				

HR, Hazard Ratio; CI, Confidence Interval.

Multivariate analyses further confirmed these findings. In the SEER cohort, 50-59 years (HR=5.93, p=0.006) and ≥70 years (HR=10.96, p<0.01), lymph node metastasis at stage ypN1-N3, and tumor stage ypT3-4 were identified as significant independent predictors of clinical outcomes. In the Chinese cohort, only individuals with stage ypT3-4, cT3-4, R1/R2, and no immunotherapy had a significantly increased risk of survival.

## 4 Discussion

With the extensive use of immunotherapy in EC treatment, the latest version of the National Comprehensive Cancer Network (NCCN) guidelines (version 4.2024) clearly recommend that patients with R0 resection of EC after nCRT should be treated with adjuvant immunotherapy if there are residual tumors (non-pCR) on pathological evaluation (11). In the field of neoadjuvant therapy, several groundbreaking researches have validated the remarkable effects of combing chemotherapy with immunotherapy. The ESCORT-NEO/ NCCES01 study demonstrated that the addition of carilizumab to chemotherapy increased the pathological complete remission rate (pCR) to 28% and achieved a major pathological remission rate (MPR) of 59.1%.This outcome was notably superior to that observed in patients who received chemotherapy alone (pCR: 4.7%, p<0.001; MPR: 43.0%, p<0.001) (12). The current standard treatment for resectable EC is nCRT combined with surgery. However, the clinical value and therapeutic benefits of PORT in patients who did not receive preoperative radiotherapy remain controversial. In the context of the current rapid development of immunotherapy, the clinical benefits and potential risks of PORT need to be reassessed.

To address this crucial clinical inquiry, we conducted a dual-cohort investigation involving 733 patients with EC, integrating data from the SEER database (407 patients) and a single-center cohort in China (326 patients), to evaluate, for the first time, the clinical value of PORT after neoadjuvant therapy in the era of immunotherapy. Our study demonstrated that PORT did not significantly enhance the survival outcomes of patients who received neoadjuvant therapy. Particularly for patients diagnosed with stage ypT3-4 or ypN+ disease. The results of this study diverge significantly from those of previous clinical studies (e.g., the 20% improvement in 5-year survival reported in the SWOG 9008 trial) (14, 15).

There are several possible reasons for this discrepancy. First, previous studies were mainly based on a population of patients who did not receive neoadjuvant therapy, whereas neoadjuvant therapy significantly improved the R0 resection rate of surgery by effectively removing potential micrometastases and achieving tumor downstaging, thereby reducing the risk of recurrent metastasis (16) and possibly compensating for the role of PORT to some extent. Second, in the context of multimodal therapy, patients may have already received an intensive treatment regimen and the continuation of PORT may be beyond the patient's tolerance. Therefore, the cumulative toxicity of multimodal therapies cannot be disregarded. According to Li et al., the incidence of grade 3 or higher adverse events (AEs) in multiple treatment regimens was as high as 65% (17). This cumulative effect of treatment toxicity may not only offset the potential treatment benefits, but also negatively affect the quality of survival and overall prognosis of patients (18, 19). Furthermore, the failure of PORT to provide a survival benefit may be closely related to the radiotherapy technique. In the era of conventional 3D-CRT, a larger target area indicates that adjacent normal tissue is unnecessarily irradiated. Over-irradiation may lead to massive immune cell depletion (20), thus weakening the body's immune defense and potentially counteracting the therapeutic effects of PORT.

Notably, PORT exhibited a considerable improvement in survival rates for patients receiving immunotherapy, which may stem from the synergistic effect of radiotherapy with immunotherapy through the activation of the systemic anti-tumor immune response through various immunomodulatory mechanisms. Numerous studies have demonstrated that radiotherapy plays a tumor-killing role by mediating the synergistic effect of CD8+ T cells with the autoimmune system (21, 22). Currently, clinical trials have confirmed the potential value of immunotherapy combined with radiotherapy. The PNEOCRTEC1901 trial (23) assessed the effectiveness and safety profile of Toripalimab combined with nCRT in treating locally advanced esophageal squamous carcinoma (ESCC). The trial, which involved 44 patients, reported an R0 resection rate of 98%, with 50% (95% CI: 35-65) achieved a pCR, and only 20% developed grade III-IV AEs. Compared to traditional nCRT, the addition of neoadjuvant immunotherapy resulted in a higher pCR rate for patients (24, 25). although there are no conclusions from large-sample, multicenter studies. Similarly, the results of a domestically conducted phase I clinical trial (PALACE-1), which further validated the efficacy of immunotherapy in the neoadjuvant phase but warned of up to 65% risk of grade 3 and higher adverse events, showed a pCR of 55.6% and R0 resection rate of 94.4% (17). These studies provide an important reference for postoperative treatment strategies. However, additional large-scale, randomized clinical studies are necessary to confirm these findings.

In terms of pathological type, the SEER cohort was predominantly AC (79.1% of cases), whereas the Chinese cohort was predominantly SCC (96.4%). This difference in biological behavior may result in differences in treatment efficacy. The CROSS study showed that after nCRT, individuals with SCC exhibited a substantially higher pCR than those with AC (49% vs. 23%, p=0.008), and the OS benefit was more significant (HR=0.48 vs. 0.74). Similarly, the NEOCRTEC5010 study confirmed a favorable response to radiotherapy in patients with SCC, with a pCR rate of 43.2% and a significant enhancement in OS (HR=0.71, 95%CI: 0.53-0.96, p=0.025) (3). These findings suggest that there are significant differences in the sensitivity of different pathological types of EC to radiotherapy (26, 27), which may explain the differences in the treatment outcomes observed in different cohorts.

Despite the limitations of our sample follow-up, we systematically assessed the toxicity characteristics across multiple treatment stages of esophageal cancer. Notably, high-quality clinical trials, such as CROSS and NEOCRTEC5010, have previously reported grade 3 or higher hematological toxicity rates of 9% and 54.3% in neoadjuvant chemoradiotherapy groups (2, 3), respectively. These significant differences may be attributed to variations in the treatment protocols, patient population characteristics, and toxicity assessment criteria. However, both studies confirmed that the overall AEs remained within an acceptable and manageable range. With the incorporation of immunotherapy in neoadjuvant treatment, we observed grade 3 or higher AEs in the 19.4%-34.1% range (12, 28), suggesting that immunotherapy did not substantially increase treatment-related risks. Although adverse reactions at individual treatment stages appeared to be relatively controlled, we must remain vigilant about the potential cumulative toxicity effects of multimodal treatments. From the perspective of PORT, while radiation-related grade 3 or higher AEs were limited to 9.5%-12.5% (29, 30), the complex cumulative toxicity in a multi-treatment context could potentially impact patient survival outcomes. Therefore, long-term safety evaluations of this treatment modality require more rigorous, large-sample systematic studies to establish definitive conclusions.

The treatment paradigm for locally advanced EC is undergoing major revolutions with the continuous development of radiotherapy and immunotherapy. Conventional radical surgical interventions are typically linked to a high incidence of complications and diminished quality of life for patients (31, 32),the "Wait and See" strategy is an innovative treatment option that has demonstrated unique advantages in specific clinical scenarios. Several clinical studies (JCOG0909, SANO) have demonstrated that an active surveillance strategy for patients with clinical complete remission (cCR) after nCRT significantly improves their quality of life and is not inferior to conventional surgery (33, 34). Key findings included the following: unnecessary surgery could be avoided in approximately 35%-52% of patients. Salvage surgery was feasible in 86% with early active surveillance, and quality of life at 6-9 months was significantly better than in the surgery group. The addition of immunotherapy has further increased the cCR rate, providing new possibilities for the "de-surgicalization" of esophageal cancer. Future studies will focus on prolonging survival while continuing to improve quality of life, which is also a great challenge for PORT.

This study has several limitations. The primary limitation is its retrospective study design, which inevitably introduces selectivity bias. Despite using PSM, some information in SEER cohort could not be collected and included in the analysis, such as patients' adverse reactions, specific regimens of immunotherapy, timing of application, dosage, and duration between neoadjuvant therapy and surgery, as well as the technical parameters and target area setting of PORT. Second, the follow-up time of the immunotherapy group was relatively insufficient; 67% and 77% of the patients receiving immunotherapy in the SEER and Chinese cohorts, respectively, were still alive and had not yet reached the median survival, which limited our assessment of the long-term efficacy of immunotherapy. In addition, although a two-cohort design was used, data from a single center in the Chinese cohort may have affected the external validity of the results. Finally, considering the significant differences in disease characteristics and treatment responses between the Eastern and Western populations, caution should be exercised when attempting to generalize the findings of this study.

In conclusion, our study provides further evidence of the effectiveness of PORT following neoadjuvant treatment in immunotherapy subgroups of patients with EC. These findings should be validated through large-scale trials.

## Data Availability

The raw data supporting the conclusions of this article will be made available by the authors, without undue reservation.
