# Supplementary material for: Survival benefits of postoperative radiotherapy in esophageal cancer during the immunotherapy era:a retrospective cohort study based on the SEER database and a single-center registry in China
Source: Front Immunol. 2025 Feb 24;16:1548520. doi: 10.3389/fimmu.2025.1548520 (PMC11891367; doi:10.3389/fimmu.2025.1548520)
Supplement: Supplementary file 1 [file DataSheet1.docx]

Supplementary Material

# Supplementary Figures and Tables

| Baseline characteristics of patients in Single-Center Cohort included in the analysis before and after PSM | | | | | | |
| --- | --- | --- | --- | --- | --- | --- |
| **Characteristics** | **Before PSM** | | | **After PSM** | | |
|  | **Non-PORT**  **（N,%）** | **PORT**  **（N,%）** | **p-value** | **Non-PORT**  **（N,%）** | **PORT**  **（N,%）** | **p-value** |
| **Total** | 229(70.2) | 97(29.8) |  | 97(50) | 97(50) |  |
| **cT stage** |  |  | 0.1218 |  |  | 0.4989 |
| cT1-2 | 38 (16.59) | 15 (15.46) |  | 13 (13.40) | 15 (15.46) |  |
| cT3-4 | 177 (77.29) | 81 (83.51) |  | 82 (84.54) | 81 (83.51) |  |
| cTx | 14 (6.11) | 1 (1.03) |  | 2 (2.06) | 1 (1.03) |  |
| **cN stage** |  |  | 0.0224 |  |  | 0.7857 |
| cN0 | 4 (1.75) | 2 (2.06) |  | 2 (2.06) | 2 (2.06) |  |
| cN1 | 65 (28.38) | 16 (16.49) |  | 11 (11.34) | 16 (16.49) |  |
| cN2 | 77 (33.62) | 27 (27.84) |  | 36 (37.11) | 27 (27.84) |  |
| cN3 | 83 (36.24) | 52 (53.61) |  | 48 (49.48) | 52 (53.61) |  |
| **resection status** |  |  | 0.0902 |  |  | 0.1518 |
| R0 | 180 (78.60) | 67 (69.07) |  | 82 (84.54) | 73 (75.26) |  |
| no-R0 | 49 (21.40) | 30 (30.93) |  | 15 (15.46) | 24 (24.74) |  |

| Baseline characteristics of patients in SEER Cohort included in the analysis before and after PSM | | | | | | |
| --- | --- | --- | --- | --- | --- | --- |
| **Characteristics** | **Before PSM** | | | **After PSM** | | |
|  | **Non-PORT**  **（N,%）** | **PORT**  **（N,%）** | **p-value** | **Non-PORT**  **（N,%）** | **PORT**  **（N,%）** | **p-value** |
| **Total** | 333(81.8) | 74(18.2) |  | 74(50) | 74(50) |  |
| **cT stage** |  |  | 0.226 |  |  | 0.586 |
| cT1-2 | 12 (3.60) | 0 (0.00) |  | - | - |  |
| cT3-4 | 33 (9.91) | 9 (12.16) |  | 6 (8.11) | 9 (12.16) |  |
| cTx | 288 (86.49) | 65 (87.84) |  | 68 (91.89) | 65 (87.84) |  |

Univariable and multivariable Cox regression analyses for overall survival of patients in Single-Center Cohort after PSM.

| **Characteristics** | **Univariate** | | | **Multivariate** | | |
| --- | --- | --- | --- | --- | --- | --- |
|  | **HR/CI** | | **p-value** | **HR/CI** | | **p-value** |
| **cT stage** |  |  |  |  |  |  |
| cT1-2 | 1 | |  | 1 | |  |
| cT3-4 | 2.93(1.56-5.5) | | 0.001 | 2.85(1.37 - 5.91) | | 0.005 |
| cTx | 7.76(2.12-28.39) | | 0.002 | - | | - |
| **cN stage** |  |  |  |  |  |  |
| cN0 | 1 | |  | 1 | |  |
| cN1 | 0.74(0.24-2.26) | | 0.597 | 1.15(0.34 - 3.92) | | 0.821 |
| cN2 | 1.12(0.4-3.15) | | 0.834 | 1.9(0.58 - 6.15) | | 0.286 |
| cN3 | 1.3(0.47-3.61) | | 0.618 | 2.35(0.7 - 7.95) | | 0.169 |
| **resection status** |  |  |  |  |  |  |
| R0 | 1 | |  | 1 | |  |
| no R0 | 2.65(1.76-3.99) | | <0.01 | 2.44(1.49-3.99) | | <0.01 |

Univariable and multivariable Cox regression analyses for overall survival of patients in SEER Cohort after PSM.

| **Characteristics** | **Univariate** | | | **Multivariate** | | |
| --- | --- | --- | --- | --- | --- | --- |
|  | **HR/CI** | | **p-value** | **HR/CI** | | **p-value** |
| **cT stage** |  |  |  |  |  |  |
| cTx | 1 | |  | 1 | |  |
| cT1-2 | - | | - |  | |  |
| cT3-4 | 1.78(0.95-3.34) | | 0.072 |  | |  |

## Supplementary Figures


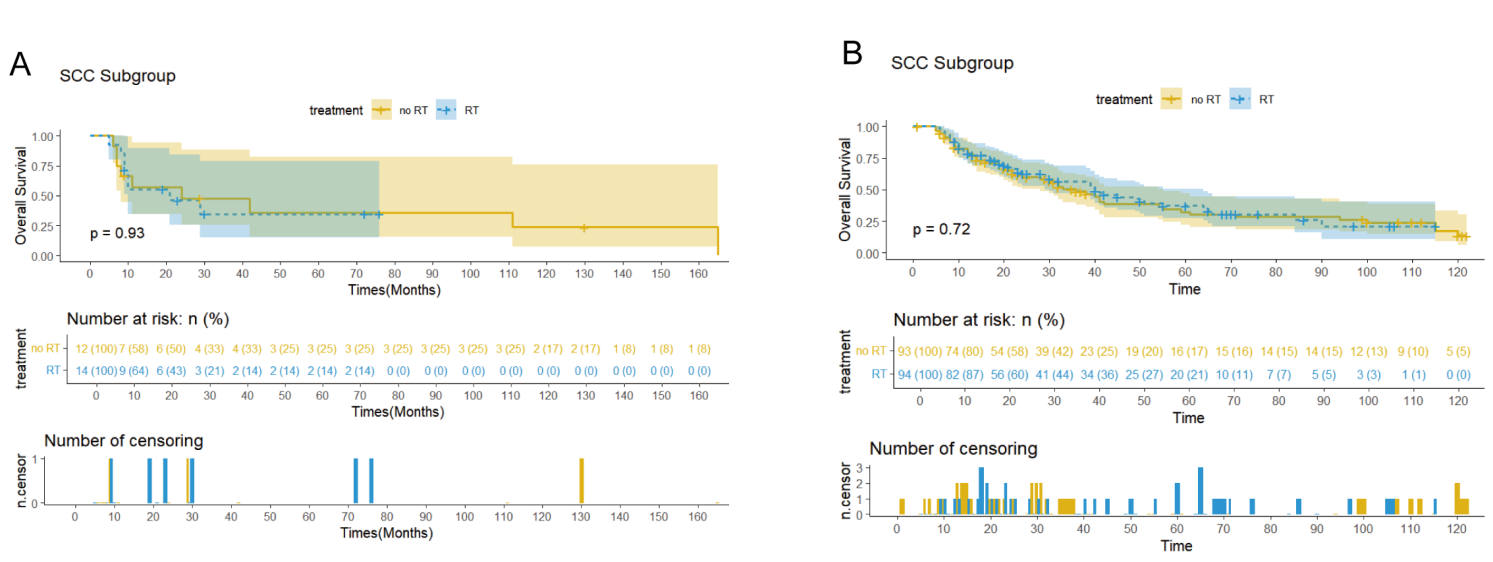


**Supplementary Figure 1.** Survival curves showed the OS for patients with squamous cell carcinoma in the SEER (A) and Chinese (B) cohorts.


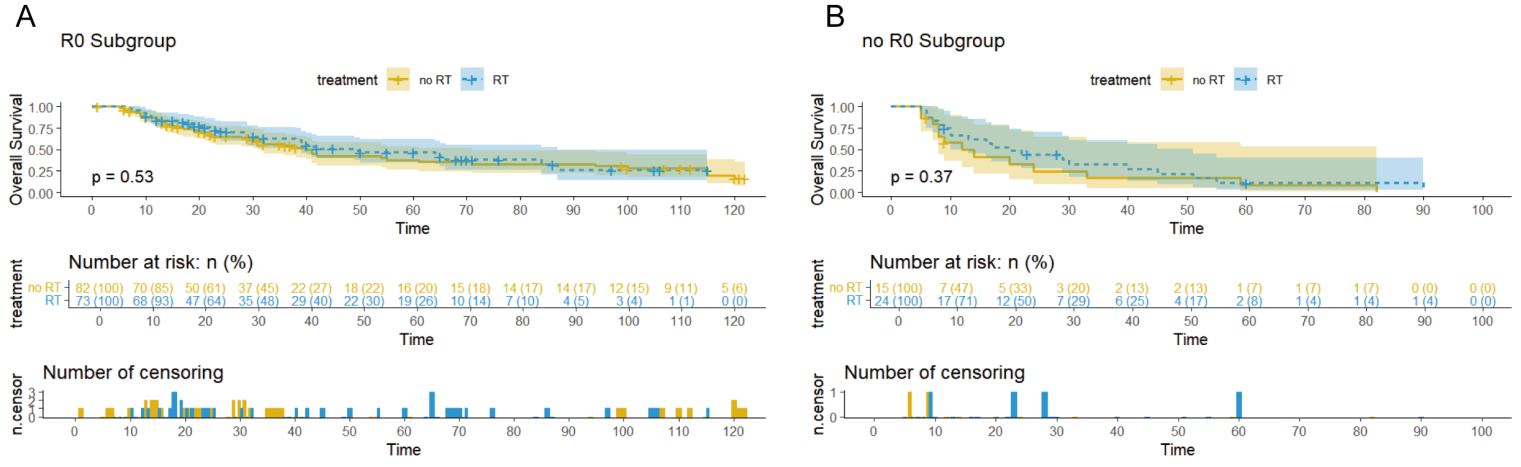


**Supplementary Figure 2.** Survival curves showed the OS for patients with R0 (A) or no R0 (B) in Chinese cohort.
